# Physical confinement promotes mesenchymal *trans*-differentiation of invading transformed cells *in vivo*

**DOI:** 10.1016/j.isci.2022.105330

**Published:** 2022-10-13

**Authors:** Teresa Zulueta-Coarasa, John Fadul, Marjana Ahmed, Jody Rosenblatt

**Affiliations:** 1 The Randall Centre for Cell and Molecular Biophysics, School of Basic and Medical Biosciences, Faculty of Life Sciences and Medicine, School of Cancer and Pharmaceutical Sciences, King’s College London, London, SE1 1UL, UK

**Keywords:** Cell biology, Organizational aspects of cell biology, Cancer

## Abstract

Metastasis is tightly linked with poor cancer prognosis, yet it is not clear how transformed cells become invasive carcinomas. We previously discovered that single KRas^V12^-transformed cells can invade directly from the epithelium by basal cell extrusion. During this process, cells de-differentiate by mechanically pinching off their epithelial determinants, but how they *trans*-differentiate into a migratory, mesenchymal phenotype is not known. Here, we demonstrate that basally extruded KRas^V12^-expressing cells become significantly deformed as they invade the zebrafish body. Decreasing the confinement that cells experience after they invade reduces the percentage of KRas^V12^ cells that *trans*-differentiate into mesenchymal cell types, while higher confinement increases this percentage. Additionally, increased confinement promotes accumulation of internal masses over time. Altogether, our results suggest that mechanical forces drive not only de-differentiation of KRas^V12^-transformed epithelial cells as they invade but also their re-differentiation into mesenchymal phenotypes that contribute to distant metastases.

## Highlights

• Transformed cells invade by basal extrusion and deform as they migrate
• Deformation of cells and their nuclei promotes mesenchymal *trans*-differentiation
• Increasing confinement and cell deformation results in more internal cell masses

## Graphical abstract

**Figure:**
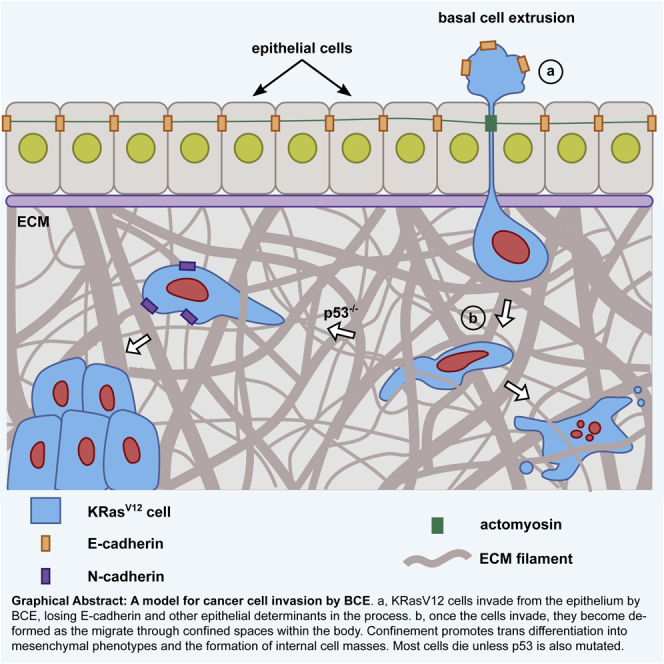

## Introduction

Although metastasis is the predominant cause of mortality in patients with cancer, how tumor cells invade to form metastases is not well understood. For a cancer cell to metastasize, it must first invade from sites where most solid tumors originate, epithelia, and then *trans*-differentiate to acquire a malignant phenotype. The prevailing metastasis model suggests that as cells accumulate mutations, they first form primary masses from which they later escape by downregulating epithelial-specific genes to undergo an epithelial-to-mesenchymal Transition (EMT) (Thiery, 2002). Transition from epithelia to mesenchymal phenotypes allows cells to dissociate from the primary tumor so that they can invade and colonize distant organs. Because these invading cells have stem-like qualities and markers that allow them to proliferate, survive, and transform into a spectrum of different cell types, the term epithelial-to-mesenchymal plasticity (EMP) has become a better descriptor and more widely used (Bornes et al., 2021).

We have shown that oncogenic KRas mutations that drive aggressive tumors (Aviel-Ronen et al., 2006; Jiang et al., 2009; Neuzillet et al., 2013) induce invasion of transformed epithelial cells by hijacking cell extrusion (Fadul et al., 2021; Slattum et al., 2014), a process that epithelia normally use to promote cell death (Rosenblatt et al., 2001). In epithelial cell extrusion, a basal intercellular actomyosin cable contracts to squeeze one cell apically from the layer to die (Eisenhoffer et al., 2012; Rosenblatt et al., 2001). KRas mutations disrupt apical extrusion, causing cells to accumulate into masses or aberrantly extrude basally, underneath the epithelium (Fadul et al., 2021; Slattum et al., 2014). Importantly, using zebrafish epidermis as a model for simple epithelia where carcinomas form, we previously found that transformed cells basally extrude directly from the epithelium, at sites independent to where they form masses (Fadul et al., 2021). Basal cell extrusion (BCE) enables KRas-transformed cells to invade, divide, and migrate throughout the zebrafish body. While most invading cells die, they can survive to form large internal masses if the tumor suppressor p53 is absent or mutant, as commonly occurs in aggressive cancers (Fadul et al., 2021). Importantly for this study, some invading KRas^V12^-expressing cells *trans*-differentiate into mesenchymal phenotypes, with a smaller, but significant fraction adopting a neuronal-like morphology (Fadul et al., 2021). The mechanisms that regulate differential fates of invaded cells are unknown.

In contrast to previous EMT models, we previously found that de-differentiation of invading transformed epithelial cells occurs suddenly and mechanically as it invades by BCE by pinching off the apical membrane containing E-cadherin and other proteins essential for epithelial identity and function (Fadul et al., 2021). Whereas all basally extruded cells lack E-cadherin, only a fraction express the mesenchymal markers N-cadherin and snail1b, suggesting a two-step model for EMT whereby cells de-differentiate by BCE and some *trans*-differentiate into mesenchymal cell types via another mechanism (Fadul et al., 2021). Given that cells can invade directly from the epithelium, the primary tumor is unlikely to influence the mesenchymal *trans*-differentiation. However, mechanical stress can induce EMT during embryonic development and tumorigenesis (Fernandez-Sanchez et al., 2015b; Gjorevski et al., 2012), suggesting a potential role for mechanical strain promoting mesenchymal-transformed cell differentiation as cells migrate throughout the tight confines of extracellular matrix and organs within the body. Here, we test the role of mechanical strain on the fate of transformed cells *in vivo* by altering the microenvironment that cells expressing EGFP-KRas^V12^ encounter as they invade and migrate throughout the body of zebrafish.

## Results

### Transformed cells deform following invasion

**Figure 1:**
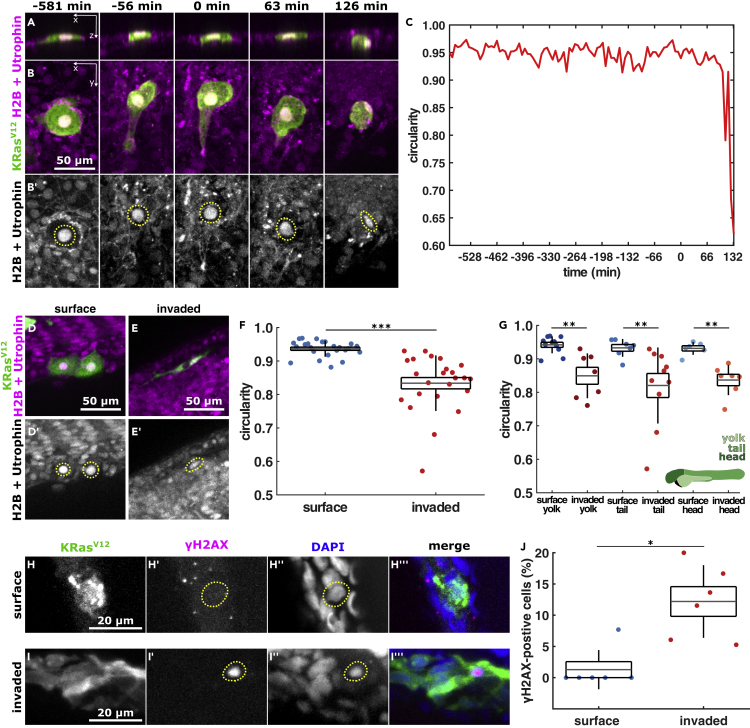
KRas^V12^ cell nuclei deform after they invade (A and B) Orthogonal and projection views of an invading cell expressing EGFP-KRas^V12^ (A, B) and H2B-RFP and mCherry-Utrophin (B′) in XZ (A) or XY (B), with time before and after BCE indicated. Scale bar, 50 μm C, nuclear circularity over time for the cell shown in A, B. D, E, example cells expressing EGFP-KRas^V12^ (D and E) and H2B-RFP and mCherry-Utrophin (D′, E′) in the outer epithelium (D) or invaded inside the embryo (E), quantified in F & G. Scale bars, 50 μm F, nuclear circularity in surface (n = 25) and invaded (n = 24) KRas^V12^ cells. (G) Nuclear circularity of surface and invaded KRas^V12^ cells located in the yolk (*n*_*surface*_ = 11 and *n*_*invaded*_*=* 7), the tail (*n*_*surface*_ = 7 and *n*_*invaded*_*=* 10) and the head (*n*_*surface*_ = 7 and *n*_*invaded*_*=* 7) of the embryo, where the diagram denotes the different regions classified. (H and I) EGFP-KRas^V12^ cells in an embryo stained for GFP, phospho-histone H2A.X, and DAPI located on the surface (H) or inside the embryo (I). Scale bars, 20 μm. (J) Percentage of KRas^V12^-invaded cells expressing phospho-histone H2A.X in the embryonic surface (n = 6) and inside the embryo (n = 6). In A, B, D, E, H, I, dotted lines outline the cell nucleus. In F, G, J, the error bars are SD(SD), the box the SEM(SEM), and the gray lines, the mean. ∗p < 0.05, ∗∗p < 0.01, ∗∗∗p < 0.001. See also Video S1.

To investigate the role of the mechanical microenvironment in promoting cell invasion, we mosaically expressed krt4:EGFP-KRas^V12^ in the outer zebrafish epidermal layer by injecting embryos with a plasmid and a p53 morpholino (MO) to enable invading cell survival. Unless otherwise indicated, all our experiments were quantified at 2 days post-fertilization (dpf). To determine if the tight confinement that invading cells encounter causes mechanical deformation of EGFP-KRas^V12^ cells, we measured the circularity of their nuclei before and after BCE in embryos expressing mCherry fused to the actin-binding domain of utrophin (Krens et al., 2017) along with H2B-RFP mRNA to visualize nuclei (Megason and Fraser, 2003). We found that the circularity of the nucleus in KRas^V12^-expressing cells decreases sharply after BCE (Figures 1A–1C and Video S1). Furthermore, the nuclear circularity of invaded KRas^V12^ cells was significantly lower than in those remaining within the embryonic surface, irrespective of their location within the body. (0.83 ± 0.01 versus 0.94 ± 0.01, p < 0.001, p < 0.01, Figures 1D–1G). The changes in nuclear deformation following invasion suggest that invading cells become confined as they migrate through the dense tissue of the zebrafish body. When cancer cells migrate through confined spaces, nuclear deformation can result in nuclear envelope rupture and DNA damage (Denais et al., 2016; Hatch and Hetzer, 2016; Irianto et al., 2017; Raab et al., 2016). Moreover, compression-derived DNA damage in breast cancer cells results in a snail1-dependent invasive phenotype (Nader et al., 2021). We sought to investigate if nuclear deformation results in DNA damage in invading EGFP-KRas^V12^ cells by immunostaining with the marker phospho-Histone H2A.X. We found that only 1.3 ± 1.3% of KRas^V12^-transformed cells in the surface of the embryo had nuclear damage, while 12.23 ± 2.4% of invaded KRas^V12^-positive cells do so (p < 0.05, Figures 1H–1J). These data suggest that the nuclear deformation cells experience during invasion and migration can damage their nuclei.

Video S1. KRasV12 cell nuclei deform after they invade, related to Figure 1A–1C

### Reduced ECM density results in fewer KRas^V12^ mesenchymal cells

**Figure 2:**
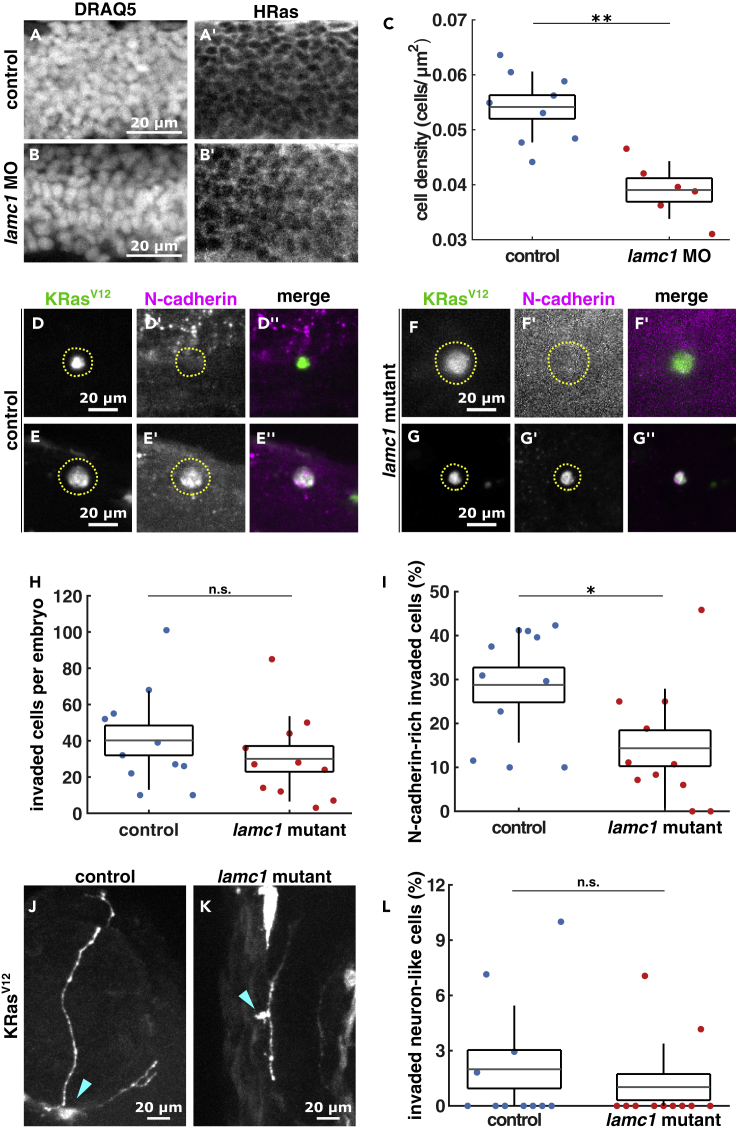
Loss of *laminin* reduces KRas^V12^ cell *trans*-differentiation into mesenchymal phenotypes (A and B) Sample images of neural tube cells expressing HRas-EGFP in control (A) and *lamc1* MO-injected (B) embryos, stained with DRAQ5 and GFP to quantify cell density in C, from 9 control embryos and 6 *lamc1* morphants. (D–G) Images of EGFP-KRas^V12^-expressing invaded cells in controls (D, E) and *lamc1* mutants (F, G) stained with GFP and N-cadherin, where the dotted lines outline cells of interest, quantified in H and I. (H) Number of invaded cells per embryo in control (n = 11) and *lamc1* mutant (n = 11) embryos. (I) Percentage of KRas^V12^-invaded cells expressing N-cadherin in controls (n = 11) and *lamc1* mutants (n = 11). (J and K) Examples of neuron-like morphology cells expressing EGFP-KRas^V12^ in control (J) and *lamc1* mutants (I), where arrowheads denote cell bodies, quantified in l. (L) Percentage of KRas^V12^-invaded cells with neuron-like morphologies in control (n = 11) and *lamc1* mutants (n = 11). In A, B, D–G, J, K, scale bars represent 20 μm. In C, H, I, L, the error bars indicate the SD, the box the SEM, and the lines, the mean. n.s., not significant, ∗p < 0.05, ∗∗p < 0.01. See also Figure S1.

Since we previously found that invading cells could differentiate into mesenchymal and neuronal-like cells following invasion (Fadul et al., 2021), we next tested if tissue confinement could impact either cell fate. To do so, we tested whether altering the physical environment that EGFP-KRas^V12^ invading cells encounter affects their *trans*-differentiation into different cell types. To reduce the extracellular matrix density within embryos (Haeger et al., 2014), we disrupted a key ECM component, laminin, using *lamc1* mutants that produce no detectable laminin (Kettleborough et al., 2013) or *lamc1* morpholinos that significantly reduce laminin expression (Parsons et al., 2002). *Lamc1* mutants have more apoptotic cells in the trunk (Parsons et al., 2002), which could further reduce cell density and confinement. To assess if laminin loss results in reduced overall cell density, we quantified neural tube cell density of control versus *lamc1* MO embryos using HRas-EGFP to define cell outlines and DRAQ5 to visualize cell nuclei (Figures 2A and 2B). We found that *lamc1* morphants had 28% reduced neural tube cell density compared to controls (p < 0.01, Figure 2C). To investigate if decreasing cell density and confinement affects *trans*-differentiation, we stained control and *lamc1* mutant embryos with the mesenchymal marker N-cadherin (Figures 2D–2G). The number of invaded cells per embryo did not change between controls (40.2 ± 8.2) and *lamc1* mutants (30 ± 7.1, p > 0.05, Figure 2H). However, 28.8 ± 4.0% of invaded KRas^V12^/p53MO cells in control embryos express N-cadherin, while only 14.4 ± 4.1% of cells invading in *lamc1* mutants do so (p < 0.05, Figure 2I). Similarly, *lamc1* morphants reduced the number of N-cadherin-positive KRas^V12^ cells by 77%, compared to controls (p < 0.05, Figures S1A–S1E), despite an 81% increase in the number of invaded cells per embryo in these morphants compared to controls (p < 0.01, Figure S1F). Interestingly, reduced confinement did not significantly alter the proportion of invaded KRas^V12^/p53MO cells that adopt neuron-like morphologies: 1.1 ± 0.7% in lamc1 mutants compared to 2.0 ± 1.1% in controls (p > 0.05, Figures 2J–2L). These data suggest that an intact ECM contributes to *trans*-differentiation of transformed cells into mesenchymal but not neuron-like phenotypes.

### Increased confinement promotes *trans*-differentiation of mesenchymal cell types

**Figure 3:**
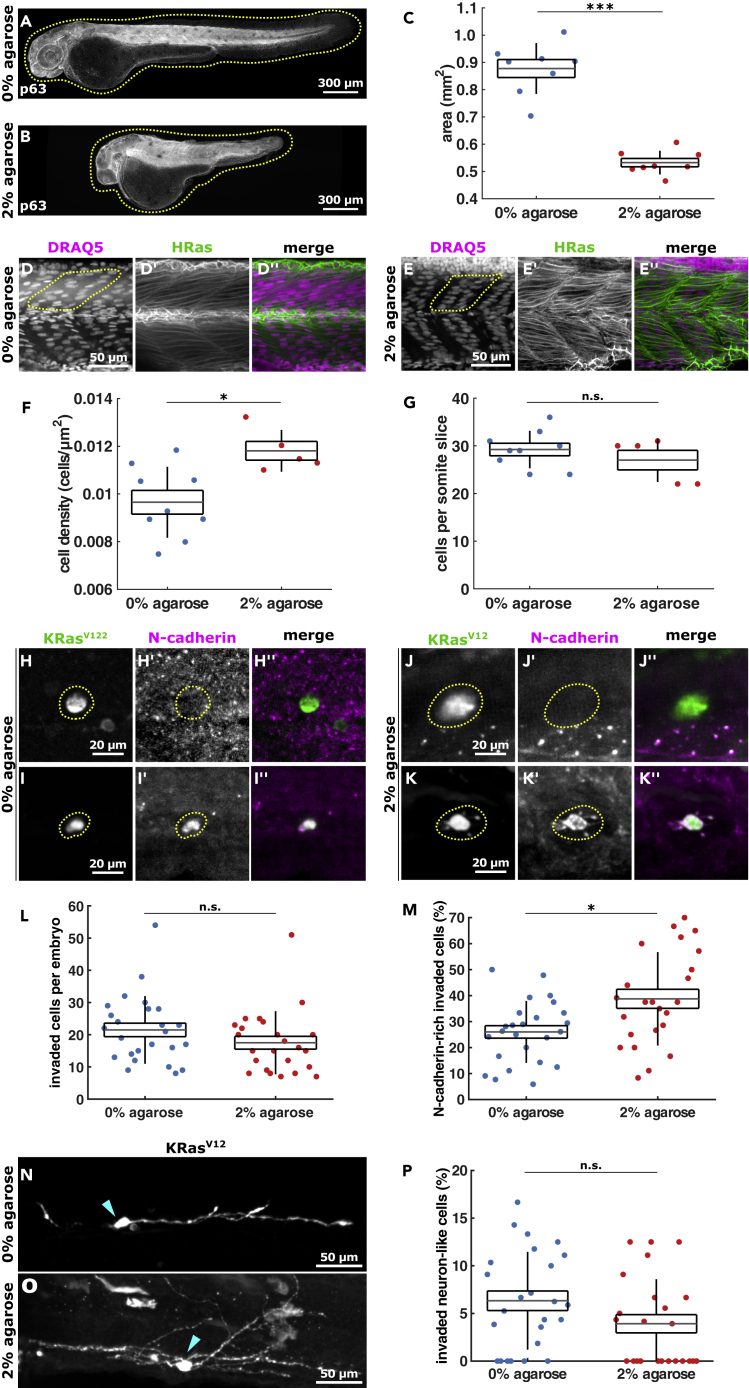
Embryo confinement in agarose promotes KRas^V12^ cell *trans*-differentiation into mesenchymal phenotypes (A and B) Example 2 dpf embryos stained for p63 that were grown from 1 to 2 dpf in 0% (A) or 2% agarose (B), with dotted lines outlining embryos. Scale bars, 300 μm. (C) Area of 2 dpf embryos embedded in 0% (n = 8) or 2% agarose (n = 8) from 1 to 2 dpf. (D and E) Somite cells expressing HRas-EGFP in embryos grown in 0% (D) and 2% (E) agarose, with dotted lines demarking half somites. Scale bars, 50 μm. (F) Somite cell density in control (0% agarose) embryos (n = 9) and embryos grown in 2% agarose (n = 5). (G) Number of cells per somite (in xy slice) in embryos grown in 0% agarose (n = 9) or 2% agarose (n = 5). (H–K) Examples of invaded EGFP-KRas^V12^-expressing cells (highlighted in dotted line) in embryos embedded in 0% (H, I) and 2% (J, K) agarose stained with GFP and N-cadherin. Scale bars, 20 μm. (L) Number of invaded cells per embryo in controls (n = 25) and 2% agarose embryos (n = 24). (M) Percentage of KRas^V12^-invaded cells expressing N-cadherin from embryos grown in 0% (n = 25) and 2% (n = 24) agarose. (N and O) Examples of EGFP-KRas^V12^ cells with a neuron-like shape in embryos grown in 0% (N) and 2% (O) agarose, where arrowheads denote cell body. Scale bars, 50 μm. (P) Percentage of KRas^V12^-invaded cells adopting a neuron-like morphology in 0% (n = 25) and 2% (n = 24) agarose. In C, F, G, L, M, P, the error bars are SD, the box, SEM, and the lines denote mean. n.s., not significant, ∗p < 0.05, ∗∗∗p < 0.001.

Because decreasing the ECM density could affect cell fate by altering signaling via reduced ECM-cell adhesion, we manipulated the mechanical microenvironment independently of ECM composition. To increase physical confinement experienced by KRas^V12^-expressing cells following invasion, we embedded embryos from 1 to 2 dpf in 2% agarose to compress them. Agarose-based confinement resulted in significantly smaller embryos, where the total embryo size (area) in 2% agarose (physical confinement) was 0.53 ± 0.01 mm^2^ (Figures 3B and 3C) compared to 0.88 ± 0.03 mm^2^ in 0% agarose (unrestricted growth, p < 0.001, Figures 3A and 3C). Embryos grown in 2% agarose also developed crooked tails (Figure 3B) and thinner somites along the anterior-posterior axis (Figures 3D and 3E), suggesting that agarose compressed the tissue during embryonic development. To determine if smaller embryos result from compression or from defects in cell proliferation, we analyzed the cell density of somites in embryos raised in 0 versus 2% agarose expressing HRas-EGFP for cell boundaries and stained with DRAQ5 to visualize cell nuclei. Development in 2% agarose increased the somite cell density by 22%, compared to control embryos (p < 0.05, Figures 3D–3F) but did not affect the number of cells per somite (p > 0.05, Figure 3G), suggesting that agarose causes physical compression, rather than reduced proliferation. To test if increasing confinement affects invading KRas-transformed cell fate, we compared their *trans*-differentiation into mesenchymal and neuronal-like cells in embryos developing in 0% agarose and in 2% agarose. We found that the number of invaded cells per embryo remained unchanged in 0% and 2% agarose-treated embryos (21.5 ± 2.1 and 17.5 ± 2.0 respectively, p > 0.05, Figures 3H–3L). Importantly, 26.0 ± 2.4% of KRas^V12^/p53MO-invaded cells within embryos grown in 0% agarose expressed N-cadherin (Figures 3H, 3I, and 3M), whereas 38.8 ± 3.7% did so in embryos grown in 2% agarose (p < 0.05, Figures 3J, 3K, and 3M). However, no significant differences in the percentage of KRas^V12^-positive neuronal-like cells were detected between embryos treated with 0% (6.3 ± 1.0%) and 2% agarose (3.9 ± 0.9%, p > 0.05, Figures 3N–3P). Together, these results suggest that mechanical compression promotes *trans*-differentiation of KRas^V12^-invaded cells into mesenchymal but not neuronal cell types.

### Altering confinement affects internal mass formation

**Figure 4:**
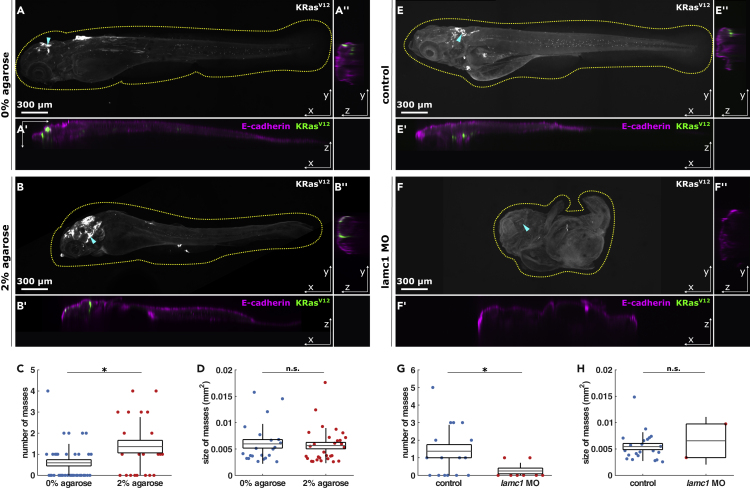
Experimental confinement promotes internal cell masses (A and B) Representative orthogonal views of 4.5 dpf embryos quantified in C & D expressing EGFP-KRas^V12^, stained for e-cadherin, and grown from 1 to 2 dpf in 0% (A) or 2% agarose (B), in XY (A, B), XZ (A′, B′) and YZ (A″, B″), with dotted lines outlining embryos and arrowheads the coordinates of XZ orthogonal sections. Scale bars, 300 μm. (C) Number of cell masses per embryo in 0% (n = 37) or 2% agarose (n = 22) embryos. (D) Cell mass areas from embryos grown in 0% (n = 22) or 2% agarose (n = 30). 4.5 dpf control (E) and *lamc1* MO-injected (F) embryos expressing EGFP-KRas^V12^, as projections (E, F), XZ (E′, F′) and YZ sections (E″, F″). Yellow dotted lines outline embryos and arrowheads mark where orthogonal sections were taken. Scale bars, 300 μm. (G) Number of cell masses per embryo in controls (n = 16) or *lamc1* morphants (n = 8). (H) Area of cell masses in control (n = 22) or *lamc1* MO-injected embryos (n = 2). In C, D, G and H, error bars are the SD, box the SEM and gray lines show the mean. n.s., not significant, ∗p < 0.05.

Embryos injected with KRas^V12^ in a *p53* mutant background develop internalized cell masses by 4.5 dpf, typically within the head (Fadul et al., 2021). The greater percentage of KRas^V12^-expressing mesenchymal cells caused by increased confinement could result in more or bigger masses by 4.5 dpf. However, compression could also cause invading cells to die instead of contributing to these masses. To investigate if mechanical induction of *trans*-differentiation affects the formation of internal masses, we embedded KRas^V12^/p53MO-injected embryos in 0% or 2% agarose from 1 to 2 dpf and fixed them at 4.5 dpf. We found that both types of embryos developed cell masses underneath the E-cadherin-stained epithelium (Figures 4A and 4B). Importantly, embryos grown in high confinement developed more internal masses than embryos grown in 0% agarose (1.4 ± 0.3 vs. 0.6 ± 0.2 masses per embryo respectively, p < 0.05, Figures 4A–4C). However, the size of the masses remained unaffected in 2% agarose embryos (0.006 ± 6.08 × 10^−4^ mm^2^) compared to controls (0.006 ± 8.05 × 10^−4^ mm^2^, p > 0.05, Figure 4D). These data suggest that mechanically increasing the percentage of KRas^V12^-expressing cells that *trans*-differentiate into mesenchymal phenotypes results in more internal masses by 4.5 dpf. To test if reducing confinement affects the later development of internal masses, we compared the number of cell masses in control KRas^V12^/p53MO-injected embryos, and embryos also injected with *lamc1* MO. *Lamc1* morphants developed fewer internal cell masses than control embryos (0.2 ± 0.1 vs. 1.3 ± 0.3 masses per embryo, respectively, p < 0.05, Figures 4E–4G). Although there were fewer masses within *lamc1* morphants, the sizes of these masses did not vary to those seen in controls (0.006 ± 3.2 × 10^−3^ mm^2^ in lamc1 to 0.005 ± 5.81 × 10^−4^ mm^2^ in controls p > 0.05, Figure 4H). However, the low number of masses in *lamc1* morphants makes statistical comparison difficult. Altogether, these results suggest that confinement has a role in the formation of internal cell masses after invasion.

## Discussion

Our results suggest that the mechanical deformation that invading transformed cells experience while migrating through confined environments promotes their *trans*-differentiation into mesenchymal cell types and internalized cell masses. Based on our current and previous findings (Fadul et al., 2021), we suggest that physical forces contribute to cell invasion and formation of distant metastases in two steps: first, apically localized epithelial determinants are pinched off during BCE to de-differentiate epithelial cells (Graphical Abstract, a); second, confinement pressure from cells migrating through tight spaces causes the de-differentiated cells to differentiate into mesenchymal cell that contribute to distant metastases (Graphical Abstract, b).

Our data imply that KRas^V12^-invading cells experience DNA damage as they migrate. Previous studies found that DNA damage can cause a partial EMT phenotype in breast cancer cells by the upregulation of snail1 (Nader et al., 2021). Our previous work showed that a fraction of transformed cells express *snail1b* in our zebrafish system, suggesting a similar mechanism (Fadul et al., 2021). Altogether, these results suggest that as invading cells migrate, DNA compression and possibly damage could promote *trans*-differentiation of KRas^V12^ cells into mesenchymal phenotypes. However, as most cells expressing phospho-Histone H2A.X have invaded, we cannot currently rule out that DNA damage promotes invasion by BCE. Future experiments using live imaging will help to distinguish between both possibilities.

Interestingly, increasing or decreasing the confinement that transformed cells experience only affects their *trans*-differentiation into mesenchymal but not neural-like cells. It is not clear what impacts the fate of invaded transformed cells with neuronal morphologies. Our previous studies suggested that they can sometimes migrate along normal neurons so local environmental signals may instead contribute to the *trans*-differentiation of these rarer cell types. Additionally, other internalized KRas^V12^/p53^-^ cells may differentiate into different unidentified cell types, making it unclear how confinement affects their differentiation.

Furthermore, increasing or decreasing confinement within the body results in more or less internal masses, respectively, by 4.5 dpf, despite the number of invaded cells at 2 dpf remaining the same with each treatment. This indicates that the mechanical forces that cells encounter as they migrate contribute to distant mass formation. Interestingly, increased confinement affects the number, but not size, of cell masses. This could suggest that compression does not affect cell proliferation but does increase oncogenicity. In our system, increasing confinement for just one day resulted in more masses over the long-term, implying that sustained confinement is not necessary for increased KRas^V12^ cell metastasis. Because most invaded cells die by 4.5 dpf in embryos in a wild-type p53 background (Fadul et al., 2021), the physical forces cells encounter and nuclear damage may contribute their death. However, when p53 is absent and cell death programs are hindered, these same physical forces could instead promote their *trans*-differentiation into mesenchymal cell types. In this way, the confined migrating cells encounter could act as a mechanical gymnasium, selecting for survival of the most aggressive, more mesenchymal-like, cells to survive.

Our findings in zebrafish may support a role for compaction forces in tumor cell *trans*-differentiation and survival into metastases in mammals, as multiple studies have highlighted a role for stiff micro-environments promoting cancer progression (Broders-Bondon et al., 2018; Butcher et al., 2009). For example, at early stages of tumorigenesis, the pressure from tumor hyperproliferation induces expression of the beta-catenin pathway in the surrounding mouse colon, transforming healthy cells into cancer cells (Fernandez-Sanchez et al., 2015a). On the other hand, fibrosis occurring at later stages of disease progression increases tumor environment stiffness that also promotes EMT (Ghajar and Bissell, 2008; Paszek et al., 2005; Weaver et al., 1996, 1997). Recent discoveries have also shown that the cell nucleus can sense shape changes caused by environmental confinement, causing the cell to adapt its response to surroundings (Lomakin et al., 2020; Venturini et al., 2020). Our findings suggest that confinement pressure directly transforms de-differentiated epithelial cells into mesenchymal phenotypes that promote their survival into distant metastases.

### Limitations of the study

While our zebrafish model allows us to follow all invading cells within a single organism, it is important to acknowledge that the signals and forces may be different from those encountered in human cancers. Admittedly, zebrafish embryos may have more embryonic signaling, which could allow for more plasticity than seen in adult cancers. In zebrafish, invading transformed cells can migrate through the tight confines of somites and collagen spikes that form fins, forces they would not encounter in human organs. Yet, it is interesting that most (but not all) masses accumulate in the head, which is likely softer tissue (Swift et al., 2013). Additionally, the manipulations we used to alter transformed cell confinement (*lamc1* mutants and 2% agarose treatment) could impact cells more broadly, by affecting signaling or developmental morphogenesis, for example. Therefore, we cannot rule out the role that non-mechanical factors might play in the changes to KRas^V12^ cell mesenchymal *trans*-differentiation we observe.

## STAR★Methods

### Key resources table

**Table:** 

REAGENT or RESOURCE	SOURCE	IDENTIFIER
**Antibodies**		

Chicken α-GFP	Abcam	Cat. # ab13970 RRID: AB_300798
Rabbit α-Tp63	GeneTex	Cat. # GTX124660 RRID: AB_11175363
Mouse α-E-cadherin	BD Biosciences	Cat. # 610181 RRID: AB_397580
Mouse α-N-cadherin	BD Biosciences	Cat. # 610920 RRID: AB_2077527
Rabbit α-N-cadherin	Abcam	Cat. # ab211226 RRID:
Rabbit Phospho-Histone H2A.X	Cell Signaling	Cat. # 9718 RRID: AB_2118009
Goat α-chicken IgY AlexaFluor-488	Thermo Fisher Scientific	Cat. # A11039 RRID: AB_2534096
Goat α-rabbit IgG AlexaFluor-568	Thermo Fisher Scientific	Cat. # A11011 RRID: AB_143157
Goat α-mouse IgG AlexaFluor-647	Thermo Fisher Scientific	Cat. # A21235 RRID: AB_2535804
Goat α-rabbit IgG AlexaFluor-647	Thermo Fisher Scientific	Cat. # A-21244 RRID: AB_2535812
Goat α-mouse IgG AlexaFluor-568	Thermo Fisher Scientific	Cat. # A-11004 RRID: AB_2534072

**Chemicals, peptides, and recombinant proteins**		

Paraformaldehyde, 16% (w/v), methanol-free	Thermo Fisher Scientific	Cat. # 28908 CAS: 50-00-0
Tricaine (ethyl 3-aminobenzoate methanesulfonate salt)	Sigma-Aldrich	Cat. # A5040 CAS: 886-86-2
*N*-Phenylthiourea	Merck	Cat. # P7629 CAS: 103-85-5
Phenol red	Sigma-Aldrich	Cat. # P0290 CAS: 143-74-8
Low Melting Point Agarose	Thermo Fisher Scientific	Cat. # 16520050
ProLong Gold	Molecular Probes	Cat. # P36930

**Critical commercial assays**		

SP6 mMESSAGE mMACHINE Transcription Kit	Thermo Fisher Scientific	Cat. # AM1340
NucAway Spin Columns	Thermo Fisher Scientific	Cat. # AM10070
Plasmid Mini Kit	Qiagen	Cat. # 12123
Plasmid Midi Kit	Qiagen	Cat. # 12143

**Experimental models: Organisms/strains**		

Zebrafish - AB/Tuebingen	N/A	N/A (wildtype strain)
Zebrafish - Ekkwill	N/A	N/A (wildtype strain)
Zebrafish - Tuepfel long fin	N/A	N/A (wildtype strain)
Zebrafish - Tg(actb1:mCherry–utrCH)	(Krens et al., 2017)	ZFIN ID: ZDB-TGCONSTRCT-151029-2
Zebrafish - Tg(bAct:hRas-eGFP)	(Cooper et al., 2005)	ZFIN ID: ZDB-TGCONSTRCT-070117-75
Zebrafish - *lamc1*^sa379^ mutant (sleepy)	(Kettleborough et al., 2013)	ZFIN ID: ZDB-FISH-150901-23200

**Oligonucleotides**		

Zebrafish p53 morpholino GCGCCATTGCTTTGCAAGAATTG	Gene Tools	ZFIN: ZDB-MRPHLNO-070126-7
Zebrafish *lamc1* morpholino TGTGCCTTTTGCTATTGCGACCTC	(Parsons et al., 2002) Gene Tools	ZFIN: ZDB-MRPHLNO-130305-2

**Recombinant DNA**		

*krt4:*EGFP-T2A-KRas^V12^	(Fadul et al., 2021)	N/A
H2B-RFP mRNA	(Megason and Fraser, 2003)	N/A

**Software and algorithms**		

MATLAB	Mathworks	https://uk.mathworks.com/products/matlab.html
DIPImage Toolbox	TU Delft	https://diplib.org/
SIESTA	Fernandez-Gonzalez lab, University of Toronto (Fernandez-Gonzalez and Zallen, 2011; Leung and Fernandez-Gonzalez, 2015	https://www.quantmorph.ca/software
NIS Elements Advanced Research v4.60	Nikon	https://www.microscope.healthcare.nikon.com/en_EU/products/software/nis-elements

### Resource availability

#### Lead contact

Further information and requests for resources and reagents should be directed to and will be fulfilled by the lead contact, Jody Rosenblatt (jody.rosenblatt@kcl.ac.uk).

#### Materials availability

This study did not generate new unique reagents.

### Experimental model and subject details

#### Zebrafish

All animal procedures were performed according to the UK Animal (Scientific Procedures) Act 1986 and carried out under Home Office Project Licence number PPL P946C972B, which was subject to local AWERB Committee review and Home Office approval. The following zebrafish lines were used: Ekkwill, AB/Tuebingen, Tuepfel long fin, Tg(*actb1:mCherry–utrCH*) (Krens et al., 2017), Tg(*bAct:hRas-eGFP*) (Cooper et al., 2005), and *lamc1*^*sa379*^ mutant (sleepy) (Kettleborough et al., 2013). Embryos were obtained by natural spawning and raised in E3 medium at 28.5°C. Embryos used for imaging were transferred to E3 with 0.003% N-phenylthiourea (PTU-E3, Merck) at 24 hpf to inhibit pigmentation. Embryos were used at 2 or 5 dpf, as indicated in each experiment. Sex cannot be determined before 5 dpf in zebrafish, so was not factored here.

### Method details

#### Microinjections and fluorescence sorting

Transposase and H2B-RFP mRNA were *in vitro* transcribed using the SP6 mMESSAGE mMACHINE Transcription Kit (Thermo Fisher Scientific), and purified using NucAway spin columns (Thermo Fisher Scientific). 2 nL of a 10-μL injection mix, comprised of 150 ng krt4:EGFP-T2A-KRas^V12^ DNA (Fadul et al., 2021),  200 ng transposase mRNA, 0.2 pmol *p53* morpholino (Gene Tools, 5′-GCGCCATTGCTTTGCAAGAATTG-3′), 1 μL phenol red (Sigma) in nuclease-free dH2O (Ambion), was microinjected into one-cell embryos. Some experiments were also injected with 50 pg H2B-RFP mRNA (Megason and Fraser, 2003) or 0.2 mM  *lamc1* morpholino (Gene Tools, 5′- TGTGCCTTTTGCTATTGCGACCTC-3′). Embryos were sorted for expression of transgenes at 1 dpf using a fluorescence dissection microscope.

#### Immunohistochemistry

Immunofluorescence was performed as described previously (Fadul et al., 2021). 2 and 4.5 dpf embryos were fixed in 4% paraformaldehyde, 4% sucrose, and 0.1% Triton X-100 in PBS overnight at 4°C. Embryos were incubated with primary antibodies overnight at 4C in 10% goat serum. Primary antibodies used were against chicken α-GFP (Abcam, 1:2000), rabbit α-phospho-Histone H2A.X (Cell Signalling, 1:100), rabbit α-p63 (GeneTex, 1:100), mouse α-E-cadherin (BD Biosciences, 1:200), mouse α-N-cadherin (BD Biosciences, 1:100 and Abcam, 1:100). Embryos were incubated with appropriate secondary antibodies (goat α-chicken-AlexaFluor-488, goat α-rabbit-AlexaFluor-568, or goat α-mouse-AlexaFluor-647 (ThermoFisher Scientific, at 1:200) in 10% goat serum overnight at 4 °C. Nuclei were stained with 20 μM DRAQ5 (ThermoFisher Scientific) or 1 μM DAPI for 30 min and washed twice with 0.5% PBST. Embryos were mounted in ProLong Gold (ThermoFisher Scientific) between a #1.5 glass coverslip and a microscopy slide, using electrical tape as a spacer and imaged on a Yokogawa spinning disk confocal microscope with an Andor iXon camera. Images were captured using either a dry 10x lens (NA 0.30; Nikon), a dry 20x lens (NA 0.75; Nikon) or a water-immersion 40x lens (NA 1.15; Nikon).

#### Live imaging

Live embryos were anaesthetised in MS-222 (Sigma-Aldrich), then mounted in 0.6% low-melt agarose as close as possible to the #1.5 glass coverslip within a slide chamber, covered with 0.02% tricaine in PTU-E3, and incubated in a controlled environment chamber at 28 °C. Imaging was done from 1 to 2 dpf with 7-min time intervals on a Yokogawa spinning disk confocal microscope with an Andor iXon camera with a dry 20x lens (NA 0.75; Nikon).

#### Agarose confinement

To induce compression, 1 dpf embryos were dechorionated, anaesthetised in MS-222 (Sigma-Aldrich) and embedded in 2% low-melt agarose in E3. Embryos were covered with 0.02% tricaine in PTU-E3, and raised at 28.5°C until 2dpf, when agarose was removed manually by gently scraping it with scalpels. Embryos were immediately fixed using the above protocol. Some of the embryos injected for this experiment were used as controls, covered with 0.02% tricaine in PTU-E3, and raised at 28.5°C until 2 or 4.5 dpf (0% agarose embryos).

### Quantification and statistical analysis

All our quantitative analysis was performed using SIESTA (Fernandez-Gonzalez and Zallen, 2011; Leung and Fernandez-Gonzalez, 2015), and custom scripts written in MATLAB (Mathworks) using the DIPImage toolbox (TU Delft).

#### Circularity

To quantify nuclear circularity, we used a mask manually overlaid on the nucleus edge. Nuclear circularity was determined as:

$$circularity=\frac{4\pi a}{p^{2}}$$

where a is the nuclear area and p the perimeter. Circularity is one for circles and lower than one for non-circular shapes.

#### Nuclear damage quantification

To characterize cells that presented nuclear damage in the surface of the embryo or after invading, embryos injected with EGFP-KRas^V12^/p53MO were stained for GFP, phospho-Histone H2A.X as a DNA damage marker and E-cadherin to denote the epithelium to distinguish whether KRas^V12^ cells had invaded. The number of invaded cells that were both KRas^V12^ and phospho-Histone H2A.X -positive was quantified. Percentages were calculated as the number of KRas^V12^ cells expressing phospho-Histone H2A.X, divided by the total number of invaded KRas^V12^ cells in the surface or inside the embryo.

#### Cell density

To measure neural tube cell density, z-stacks with a z resolution of 0.5 μm of embryos expressing a membrane marker (HRas-GFP) and a nuclear stain (DRAQ5) were acquired. Then the surface of the neural tube under somites 14–15 was visually detected and a substack was created from 19 μm to 21 μm underneath. A maximum Intensity Projection (MIP) was generated and the area under the somite of interest was delineated by manually overlaying a mask. Finally, the number of nuclei were counted to quantify the cell density as:

$$cell\;density=\frac{number\;of\;nuclei}{area}$$

A similar procedure was used to quantify the cell density in the somites. A substack 15 to 20 μm below the surface of somites 14–15 was created and the cell density was quantified as above.

#### Trans-differentiation quantification

To characterize mesenchymal trans-differentiation levels under different treatments, embryos injected with EGFP-KRas^V12^/p53MO were stained for GFP, N-cadherin as a mesenchymal marker and E-cadherin or p63 to delineate the epithelium or the periderm to score only KRas^V12^ cells that had invaded beneath the periderm. The number of invaded cells that were both KRas^V12^ and N-cadherin-positive was computed. Neuron-like cells expressing EGFP-KRas^V12^ were classified based in their morphology, the expression of GFP and their location beneath the periderm. Percentages were calculated as the number of KRas^V12^ cells expressing N-cadherin or neuron morphology, divided by the total number of invaded KRas^V12^ cells.

#### Fish area

To quantify the lateral area of control fish and those embedded in 2% agarose, embryos were stained with p63 to mark the periderm and obtain the embryo outline. Embryos were mounted laterally, and a z-stack of the whole embryo was acquired. MIPs of the resulting images were used to manually segment the fish outline.

#### Internal cell masses

For the analysis of number and size of internal cell masses by 4.5 dpf, we imaged z-stacks of EGFP-KRas^V12^/p53MO-injected embryos, fixed and stained with E-cadherin and GFP. To identify masses, we created binary masks from MIP of the EGFP-KRas^V12^ signal, by selecting pixels above an intensity threshold. The intensity threshold was the mean image intensity plus four standard deviations. Individual masses were labelled in the binary image and their area was quantified. Labelled objects smaller than 0.0025 mm^2^ were ignored for the analysis, to avoid including individual cells. Cells mis-expressing EGFP (Fadul et al., 2021) were also excluded from the analysis. To ensure that the labelled objects were indeed internal masses we visually confirmed that the EGFP-KRas^V12^ signal was below the E-cadherin-labelled epithelia in the z-stacks.

### Statistical analysis

To evaluate sample means, we used a non-parametric Mann–Whitney test (Glantz, 2012). To compare more than two groups, we used a Kruskal–Wallis test to reject the null hypothesis, and a Mann–Whitney test with the Holm-Sidak adjustment for pairwise comparisons. Exact value of *n* and how significance was defined, can be found in the figure legends.

## Data Availability

• All data reported in this paper will be shared by the lead contact upon request. • This paper does not report original code. • Any additional information required to reanalyze the data reported in this paper is available from the lead contact upon request.
