# Supplementary material for: Physical confinement promotes mesenchymal trans-differentiation of invading transformed cells in vivo
Source: iScience. 2022 Oct 13;25(11):105330. doi: 10.1016/j.isci.2022.105330 (PMC9618776; doi:10.1016/j.isci.2022.105330)
Supplement: Document S1. Figure S1 [file mmc1.pdf]

iScience, Volume *25*

Supplemental information

Physical confinement promotes mesenchymal *trans*-differentiation of invading transformed cells *in vivo*

Teresa Zulueta-Coarasa, John Fadul, Marjana Ahmed, and Jody Rosenblatt

Fig. S1: KRas^{V12} cells in *lamc1* morphants present lower rates of trans-differentiation, related to Fig. 2. a-d, representative EGFP-KRas^{V12}-expressing invaded cells in controls (a, b) and *lamc1* morphants (c, d), with dotted lines outlining cells of interest. Scale bars, 20 µm. **e**, percentage of KRas^{V12} invaded cells expressing N-cadherin in control (n = 20) and *lamc1* morphants (n = 19). **f**, number of invaded cells per embryo in control (n = 20) and *lamc1* MO-injected (n = 19) embryos. In **e** and **f**, the error bars indicate the s.d., the box the s.e.m., and the lines the mean *$P < 0.05$, **$P < 0.01$.

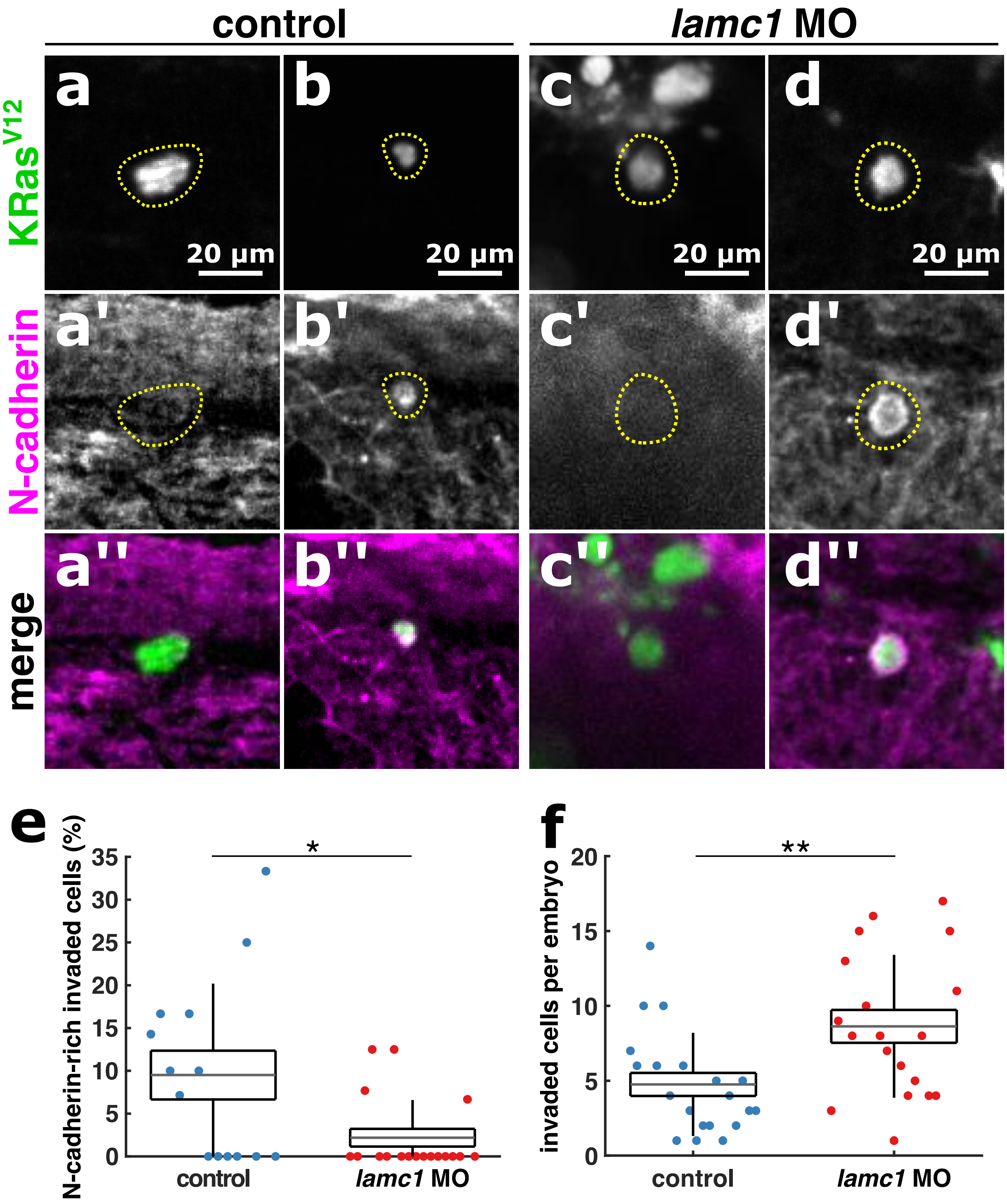

Supplementary Video 1, related to Fig. 1. The nucleus of a $KRas^{V12}$-positive cell gets deformed as it is basally extruded. A $KRas^{V12}$-positive cell (green) is basally extruded in an embryo co-expressing mCherry-utrophin and H2B-RFP (magenta). A stack was acquired every 7 min for 11 h 47 min. Time after basal extrusion is shown.
